# Scientific Advancements in Gene Therapies: Opportunities for Global Regulatory Convergence

**DOI:** 10.3390/biomedicines13030758

**Published:** 2025-03-20

**Authors:** Jimi Olaghere, David A. Williams, Jeremy Farrar, Hildegard Büning, Cecelia Calhoun, Tony Ho, Maneesha S. Inamdar, David Liu, Julie Makani, Kwasi Nyarko, Sol Ruiz, John Tisdale, Joseph M. McCune, Esther Boadi, Reagan-Udall Foundation for the FDA

**Affiliations:** 1 Sugarloaf Capital, Atlanta, GA 30309, USA; 2 Boston Children’s Hospital, Boston, MA 02115, USA; 3 Harvard Medical School, Boston, MA 02115, USA; 4 World Health Organization, Avenue Appia 20, 1211 Genève, Switzerland; 5 Hannover Medical School, 30625 Hannover, Germany; 6 School of Medicine, Yale University, Orange, CT 06477, USA; 7 Pivotal Lifesciences, Cambridge, MA 02142, USA; 8 Institute for Stem Cell Science and Regenerative Medicine, Bangalore 560065, India; 9 Broad Institute of Massachusetts Institute of Technology (MIT) and Harvard, Cambridge, MA 02142, USA; 10 Department of Haematology and Blood Transfusion, Muhimbili University of Health and Allied Sciences (Tanzania), Dar-Es-Salaam P.O. Box 65001, Tanzania; 11 WHO Regional Office for Africa (WHO-AFRO), Cité du Djoué, Brazzaville P.O. Box 06, Congo; 12 Spanish Medicines Agency (AEMPS), C/Campezo, 1, Edificio 8, 28022 Madrid, Spain; 13 National Heart, Lung, and Blood Institute (NIH), Bethesda, MD 20892, USA; 14 Gates Foundation, Seattle, WA 98109, USA; mike.mccune@gatesfoundation.org; 15 Reagan-Udall Foundation for the FDA, Washington, DC 20036, USA

**Keywords:** gene therapy, low-and middle-income countries (LMICs), sickle cell disease (SCD), clustered regularly interspaced short palindromic repeats (CRISPR), chimeric antigen receptor T cell (CAR T cell)

## Abstract

On 4 September 2024, the Reagan-Udall Foundation for the FDA (FDA Foundation) in collaboration with the Food and Drug Administration (FDA) and the Gates Foundation hosted a workshop titled “Scientific Advancements in Gene Therapies: Opportunities for Global Regulatory Convergence”. The event brought together a diverse group of experts, including international regulatory bodies, regulated industries, healthcare professionals, patients, academic researchers and global health advocates, to discuss the rapid advancements in gene therapy and the pressing need for equitable access in low-and middle-income countries (LMICs), with sickle cell disease (SCD) serving as the model disorder for the discussions. Although there has been significant progress in gene therapy, such as breakthroughs in clustered regularly interspaced short palindromic repeats (CRISPR)-based technologies and FDA-approved therapies, access to these therapies remain limited in underresourced regions. The workshop addressed critical challenges, including the high cost of therapies, regulatory gaps and barriers and ethical concerns regarding informed consent and public engagement in LMICs. This paper highlights the critical discussion points from the workshop with a focus on exploring strategies for global regulatory convergence, the role of international collaborations and the potential pathways to making gene therapies affordable and accessible to all.

## 1. Introduction

There have been significant advancements in gene therapy, particularly in the treatment of hematological disorders, neurodegenerative diseases and some forms of cancers [1]. However, as highlighted in the “Scientific Advancements in Gene Therapies: Opportunities for Global Regulatory Convergence” workshop convened by the Reagan-Udall Foundation for the FDA on 4 September 2024, the primary focus of global gene therapy discussions has been in high-income countries, leaving low- and middle-income countries (LMICs) behind despite their heavy burden of genetic disorders like sickle cell disease (SCD). The workshop emphasized the urgent need for changes in regulatory frameworks that promote global access to gene therapies to all parts of the world, particularly in resource-limited settings such as sub-Saharan Africa.

Opening remarks provided by Dr. Peter Marks from the FDA Center for Biologics Evaluation and Research, Dr. Julie Makani from the Muhimbili University of Health and Allied Sciences and Dr. Mike McCune from the Gates Foundation emphasized that the need for global collaboration, equitable access and regulatory innovation to make gene therapies better available to patients in LMICs, particularly for highly prevalent diseases such as SCD. These remarks set the stage for a series of sessions which explored the current state of gene therapy, the regulatory challenges and the roles of industry, regulators and healthcare systems in shaping the future of the field. Provided below are summaries from each presentation, which focused on diverse viewpoints, current therapeutic strategies, challenges to global implementation and potential solutions for equitable access.

## 2. Session 1: The Current State of Gene Therapy

### 2.1. Scientific Advancements in Gene Therapies: Opportunities for Global Regulatory Convergence: The Current State of Gene Therapy, David A. Williams, MD, Boston Children’s Hospital/Harvard Medical School

**Figure 1:**
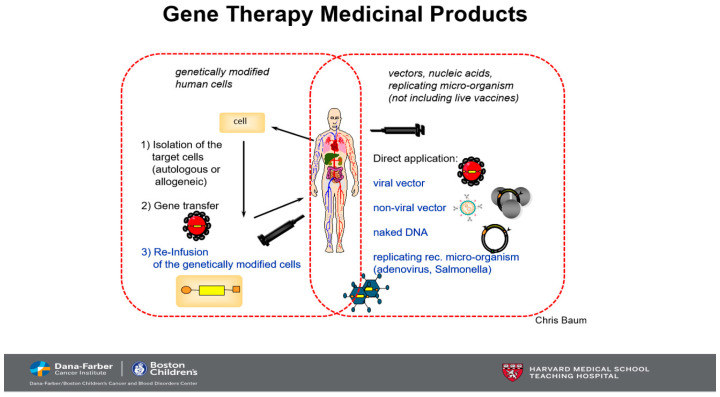
Ex vivo gene therapy involves removing cells from the body and modifying them in a GMP facility—typically using viral vectors or other methods—before reinfusing them (**left**). In contrast, in vivo gene therapy directly introduces genetic material into the body (**right**).

Gene therapy is divided into two main approaches: ex vivo and in vivo therapies (Figure 1). Ex vivo gene therapy involves the harvesting of target cells from the patient, followed by the genetic modification of the harvested cells with a gene of interest in a controlled Good Manufacturing Practice (GMP) laboratory environment. The transduced cells are then reintroduced into the patient’s body to correct the phenotype of the disease. Although this method has proven effective, ex vivo gene therapy is complex, costly and highly individualized, requiring prolonged hospital stays and intensive care. Conversely, in vivo therapy involves direct delivery of the genetic material into the patient’s body, typically via intravenous (IV) injections to target specific tissues. While in vivo therapy avoids the complexities associated with cell extraction and reinfusion, it presents challenges such as immune responses to vectors used, inefficient delivery of genetic material to targeting tissues and concerns over the persistence of gene expression [2].

A range of FDA-approved gene therapies have become available to patients, addressing conditions such as spinal muscular atrophy (SMA), hemophilia, adrenoleukodystrophy and SCD (Table 1) [3]. Chimeric antigen receptor T-cell (CAR T-cell) therapies have revolutionized the treatment landscape for cancers, such as B-cell leukemias and lymphomas, drastically improving outcomes in cases of relapsed leukemia [4]. Despite these significant advancements, the global adoption of gene therapy faces significant challenges. Barriers to global accessibility of gene therapy are multifaceted. Manufacturing complexities, particularly in ex vivo approaches, require specialized facilities and individualized products, leading to extended production timelines and a high cost of treatment. Current approaches necessitate intensive inpatient treatment, which is often unavailable in resource-limited regions. Furthermore, a global shortage of manufacturing capacity affects even high-income countries. Low and middle-income countries may need to consider outsourcing manufacturing in the short term while investing in research and development to build their own capabilities for local or centralized manufacturing in the long term. Additionally, a lack of convergence across regulatory agencies contributes to delays in therapy approvals and limits access to innovative treatments. From a financial standpoint, the high costs associated with the manufacturing of gene therapy will be difficult to sustain, even within high-income countries. Even in those nations, there is a lack of equitable access to healthcare facilities capable of administering these therapies, particularly in rural areas where tertiary care centers are sparse.

The implementation of a hub-and-spoke model for healthcare delivery may enhance the distribution of these therapies across rural populations and underresourced healthcare settings worldwide. This approach has been adopted at Boston Children’s Hospital to advance clinical development and patient care, particularly in gene therapy. The process begins by identifying a clinical physician lead and product champion to drive the initiative. A patient workflow is then established, and all relevant stakeholders are engaged early to ensure seamless care coordination. For sponsored programs, a liaison is designated to facilitate collaboration, education and communication and ensure the smooth delivery of therapy. Lastly, institutional processes are standardized, and logistical challenges such as payer support and relocation are addressed to efficiently conduct clinical trials and implement commercial therapies for patients with severe diseases. This model illustrates that early involvement of community and patient advocacy groups can aid in the successful implementation of trials, and it enables the hospital to efficiently conduct clinical trials and deliver commercial gene therapies for patients with severe diseases. Additional potential solutions include point-of-care manufacturing to produce therapies locally, reducing both costs and logistical challenges. Affordable licensing fees for LMICs and investing in the local manufacturing capacity have the potential to lower costs over time. Developing funding models that account for long-term healthcare savings and improved patient outcomes that these therapies can provide is essential in the broader adoption of such therapies.

### 2.2. Regulation of Cell and Gene Therapy in Low-And-Middle-Income Countries (LMICs): The Case of Africa, Eric Karikari-Boateng, MS, Director of the Center for Laboratory Services and Research at the Food and Drug Authority (FDA) Ghana

The current state of gene therapy in LMICs, particularly in Africa, is characterized by significant challenges related to access, infrastructure and regulatory capacity. Although regulatory definitions for cell and gene therapy are provided by major agencies such as the U.S. FDA, European Medicines Agency (EMA) and the World Health Organization (WHO), alignment across these bodies has not translated into equitable global access [5]. The translation of cell and gene therapy to LMICs, particularly in Africa, faces significant barriers, primarily due to the lack of clinical trials and limited regulatory capacity.

Between 1991 and 2008, only about 2% of the 274,000 global clinical trials were conducted in Africa, despite bearing approximately 90% of the global disease burden. By mid-2022, more than 2000 gene therapies focusing on conditions such as oncology, neurology, hematological diseases and cardiovascular diseases were in various stages of development globally. However, fewer than five clinical trials were being conducted in Africa, with research largely concentrated in Egypt and South Africa. This highlights the continent’s continued stark underrepresentation in global research efforts [6]. This underrepresentation is further compounded by a lack of regulatory frameworks, limited infrastructure and insufficient training. Only six African nations—Egypt, South Africa, Ghana, Nigeria, Tanzania and Zimbabwe—meet the WHO’s Maturity Level 3 (ML3) benchmark for regulatory capacity. Additionally, many countries in Africa lack the necessary guidance documents or regulations for cell and gene therapy, often relying on WHO guidelines that are yet to be fully developed. Clinical trials are typically regulated under biologics frameworks, which fail to address the complexities of these advanced therapies.

Moreover, region-specific research is crucial due to the genetic diversity in LMIC populations. As such, therapies developed in high-income countries (HICs) may not translate directly to African population as genetic differences can impact treatment effectiveness. Therefore, diseases such as HIV, hepatitis B, SCD, beta-thalassemia, hemophilia and various cancers remain inadequately addressed despite their significant burden. Furthermore, public and patient education on cell and gene therapy is lacking, resulting in mistrust and misconceptions about these treatments.

### 2.3. Ending Disease in Africa: Strategic Approach to Developing Capacity and Providing Regulatory Oversight for Gene Therapy Clinical Trials in Africa, Kwasi Nyarko, PhD, WHO Regional Office for Africa (WHO-AFRO)

Building on previous discussions, Africa’s participation in global clinical trials remains limited, accounting for less than 2.5% of total global activity, with gene therapy trials being even scarcer [7]. This limited involvement reflects the ongoing barriers in access to medical care, therapeutic infrastructure, ethics and regulatory oversight discussed in the preceding talks. Despite these barriers, Africa presents significant potential for gene therapies due to its large and genetically diverse population, which is crucial for advancing research into personalized gene therapies. As previously highlighted, this genetic variability is crucial for ensuring that gene therapies are effective across different populations, particularly when addressing region-specific genetic conditions.

The African Vaccine Regulatory Forum (AVAREF), established by the WHO in 2006, has played a pivotal role in improving the regulatory and ethical capacity across the continent. Initially focused on vaccines, the AVAREF has since expanded to support all medicines, including the regulation of cell and gene therapies, fostering collaboration among African nations. However, critical gaps remain, particularly in the availability of expert reviewers and the development of digital infrastructure to manage clinical trial oversight. These challenges hinder the capacity of many African countries to conduct advanced clinical trials efficiently.

Africa’s capacity for Phase I clinical trials, which is vital for early-stage therapeutic research, remains underdeveloped. As noted previously, only a few African nations have achieved WHO ML-3 using the WHO’s Global Benchmarking Tools (GBTs) for regulatory functioning. Expanding regulatory capacity across more African countries will require targeted investments in human resources and infrastructure. Multi-country joint regulatory reviews have emerged as a promising solution, enabling multiple countries to collaborate on clinical trial approvals, thereby promoting regulatory harmonization, convergence and capacity-building. The African Medicines Agency (AMA), once fully operational, is expected to further drive convergence efforts, streamlining regulatory processes and enhancing access to innovative therapies like gene therapy.

Community engagement and public education are also crucial for addressing public misconceptions and building trust in gene therapies. Misunderstandings about advanced medical therapies can lead to hesitancy, a challenge that has been seen in global vaccination efforts. By engaging Africa and its communities early in clinical trials and providing clear, accurate information, African nations can enhance public acceptance of new treatments. Additionally, including African populations in gene therapy research ensures that these therapies are tailored to the continent’s unique genetic, societal and environmental contexts, a point highlighted in earlier discussions.

To advance gene therapy in Africa, addressing the gaps in regulatory capacity and increasing public and community involvement are key steps forward. With the continent’s growing population, genetic diversity and strategic regulatory improvements, Africa has the potential to play a pivotal role in the global landscape of gene therapy, particularly through continued investment in regulatory capacity-building and convergence efforts.

### 2.4. Ethical Considerations for Gene Therapies in LMICs, Maneesha Inamdar, PhD, Director of the Institute for Stem Cell Science and Regenerative Medicine, Bengaluru, India

The ethical considerations, decision-making processes and access challenges associated with cell and gene therapy are critical to understanding their broader implications in healthcare, particularly in LMICs. While these technologies provide hope for addressing unmet medical needs, they are also met with concern regarding their potential use for health or cosmetic enhancements, rather than the treatment of serious diseases. (A recent analysis highlights the current status of gene and cell therapies in LMICs, emphasizing the need for improved access while discussing existing barriers and product development efforts in Brazil, South Africa and India [8]) Establishing clear boundaries for their application raises fundamental questions about what constitutes essential treatment versus enhancement.

Navigating these concerns necessitates an assessment of the unmet healthcare needs as defined by the WHO, which states that health encompasses “a state of complete physical, mental, and social well-being and not merely the absence of disease or infirmity”. This definition underscores the subjective and context-dependent nature of health needs, highlighting the complexities involved in determining what is truly essential in medical care.

The global distribution of gene therapy trials is disproportionately skewed toward wealthier nations, yet it is important to recognize that 8 of the 10 most populous countries are classified as LMICs, comprising approximately one-third of the world’s population. This disparity illustrates a significant gap between the global disease burden and the availability of clinical trial opportunities [8,9]. Additionally, in LMICs, government insurances primarily cover infectious diseases, making access to expensive therapies like gene therapy challenging. Despite the barriers to access and approval in different resource-limited regions, some LMICs have made progress in advancing gene therapies.

A notable example of the increased potential of gene therapy in resource-limited regions is India’s first commercially approved CAR-T therapy developed by IIT Bombay, the Tata Memorial Centre and Immuno ACT for B-cell lymphomas and B-acute lymphoblastic leukemia. The treatment costs 0.4–0.45 crores (~USD 50,000–55,000), nearly one-tenth of the cost in the United States, highlighting the impact of local innovation on the affordability of gene therapies in LMICs. Moreover, other LMICs (e.g., China, Brazil and the Philippines) have initiated programs or have received approval for gene therapies to target high-burden diseases. However, the implementation of gene therapies in LMICs present several key challenges, including scientific uncertainties related to long-term safety and unintended side effects, regulatory gaps, limited public understanding of gene therapy complexities, issues surrounding the adequacy of informed consent, cultural differences in healthcare decision-making, financial barriers and healthcare infrastructural disparities. While these therapies are recognized as scientifically feasible and ethically acceptable, challenges related to potential adverse effects and long-term safety remain.

In many LMICs, the general population may not fully grasp the complexities of gene therapies. More immediate concerns, such as basic needs, may logically take precedence over advanced medical technologies. This focus on daily survival complicates the ethical and access considerations surrounding the introduction of gene therapies in these regions. It is essential to align societal understanding of gene therapies with the scientific advancements that are taking place. Ethical considerations surrounding informed consent for somatic gene therapy encompass more than just obtaining permission; they also involve the nuances of the refusal process and assent, especially in cases involving children [10,11,12]. Variability in statutory ages of consent across regions and cultural practices influencing decision-making complicate the consent process, as patients often consult family members when making healthcare decisions.

Access to gene therapies in LMICs is further obstructed by prohibitive costs and insufficient insurance coverage for genetic conditions. This, coupled with disparities in healthcare infrastructure and uniform longitudinal care, creates significant barriers to accessing these innovative therapies [13,14].

Addressing these ethical considerations necessitates robust public engagement and education strategies tailored to local contexts. Although gene therapies may be scientifically validated, their acceptance ultimately hinges on a comprehensive understanding of societal needs and values. Integrating local perspectives into the development and implementation of gene therapy initiatives is essential to ensure that these advanced therapies resonate with the communities they aim to serve.

## 3. Session 2: Panel Discussion to Bring Additional Perspectives on Gene Therapies from Different Stakeholders

Panelists: Jimi Olaghere, Gene Therapy Recipient; David A. Williams, MD, Harvard Medical School; Eric Karikari-Boateng, MS, Food and Drugs Authority Ghana; Kwasi Nyarko, PhD, WHO Regional Office for Africa (WHO-AFRO); and Maneesha Inamdar, PhD, Institute for Stem Cell Science and Regenerative Medicine.


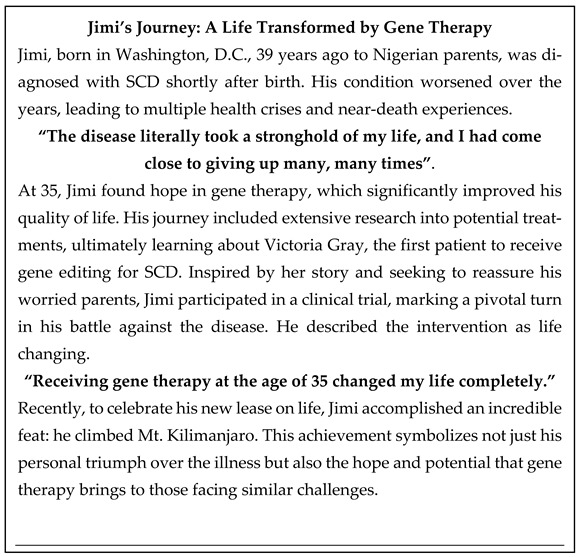


The transformative potential of gene therapy is demonstrated through the patient’s journey, which highlights the critical need for increased access to such interventions, particularly for underserved populations. The introduction of gene therapy has resulted in substantial improvements in the patient’s quality of life, alleviating both physical symptoms and the psychological burdens associated with SCD.

Key takeaways from this panel discussion are provided below, showcasing the complex challenges and opportunities within the landscape of gene therapy. The discussion emphasized the need for collaborative, culturally sensitive and ethically grounded approaches to enhance the accessibility and effectiveness of gene therapy for underserved populations.

***Key Highlights from Discussion***

- **Disparities in Access to Treatment:**

Despite advancements in gene therapy, significant disparities persist in access to these treatments, particularly in low-resource and even middle-resourced settings, where healthcare infrastructure is often lacking.

- **Need for Capacity-Building in Healthcare Systems:**

Enhancing healthcare systems is essential for the effective delivery of advanced therapies. Targeted training for healthcare providers and public education initiatives are crucial for ensuring the safe and ethical application of gene therapies.

- **Importance of Collaboration and Advocacy:**

Collaborative efforts with regional organizations are necessary to improve healthcare delivery and access to transformative therapies. Ongoing advocacy is vital to ensure that all individuals with SCD can benefit from advancements in medical treatments, regardless of their socio-economic status.

Regulatory collaboration and communication are needed to establish trust and ensure that policies are practical and effective.

- **Informed Consent and Patient Education:**

Simplifying consent materials and incorporating patient testimonials can enhance understanding and ensure that prospective participants are adequately informed about gene therapy procedures and risks.

- **Cultural Considerations:**

Tailoring approaches to consider cultural and traditional nuances is essential for patient recruitment and education. Strategies should be sensitive to local customs and values to effectively engage diverse populations.

- **Holistic Treatment Approach:**

The narrative around gene therapy as a “one-time treatment” must shift towards a holistic approach that encompasses post-treatment support. Patients transitioning from chronic illness to wellness require ongoing psychological and emotional support.

## 4. Session 3: The Next Generation of Gene Therapies

### 4.1. Editing the Genome Without Double-Strand DNA Breaks: David Liu, PhD, Broad Institute of Massachusetts Institute of Technology (MIT) and Harvard

**Figure 2:**
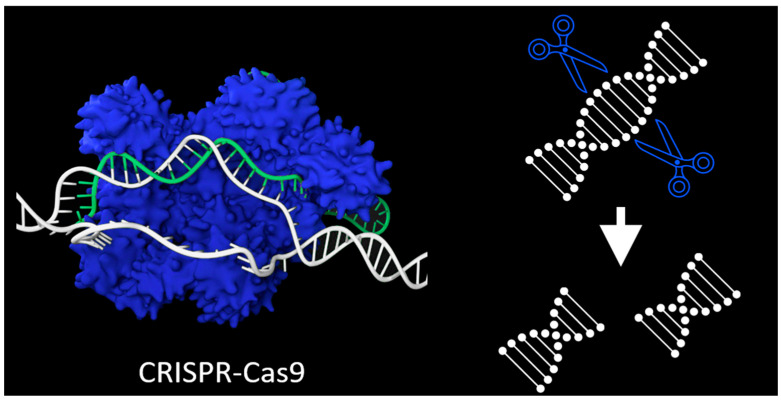
CRISPR-Cas9 is a protein (shown in blue) that acts as a powerful gene-editing tool. Guided by a small RNA segment (in green) known as the guide RNA, CRISPR-Cas9 locates a matching DNA sequence and cuts it, breaking the double helix. This precise cut can disrupt a gene, a mechanism originally evolved by bacteria to defend against viral infections.

Gene-editing technologies, particularly clustered regularly interspaced short palindromic repeats (CRISPR)-Cas9, base editors and prime editors, are transforming the treatment landscape for genetic disorders by addressing the root cause: mutations in DNA. CRISPR-Cas9, the first widely adopted gene-editing tool, works by cutting DNA at specific sites, allowing for gene disruption [15]. Clinically, this technology has been utilized in trials to treat diseases such as amyloidosis and sickle cell disease, where disruption of specific genes can lead to therapeutic benefits (Figure 2). Notably, the first FDA-approved gene-editing drug, Casgevy (exagamglogene autotemce), employs CRISPR-Cas9 to reawaken dormant hemoglobin genes in patients with sickle cell disease, marking a significant milestone in gene therapy.

While the gene-disrupting ability of CRISPR-Cas9 has proven to be effective in some cases, most genetic diseases require more precise corrections such as changing single DNA letters rather than disrupting entire genes. To address this challenge, researchers developed base editors, which are molecular machines capable of converting one DNA letter to another without breaking the DNA strand [15,16,17]. Base editors enable targeted conversion of C to T or A to G and have demonstrated the ability to correct approximately 30% of known disease-causing mutations. These tools have shown efficacy in preclinical models, correcting mutations that cause conditions like progeria, muscular dystrophy and certain genetic blindnesses. Base editors have also advanced to at least 11 distinct types, with at least four trials reporting promising outcomes. One notable case involved a 13-year-old patient with T-cell leukemia who achieved remission after receiving base-edited CAR-T cells.

However, base editors are limited in scope, primarily addressing only four of the twelve possible types of DNA base changes. To tackle the remaining mutations, prime editors were developed in 2019. Prime editing offers a more flexible approach, functioning like a “search-and-replace” tool for DNA. Unlike CRISPR-Cas9, which cuts DNA, or base editors, which correct single letters, prime editors can insert, delete or swap entire sequences of DNA, allowing for the correction of a broader range of genetic mutations [18]. Early studies demonstrated the potential of prime editing in correcting a variety of disease-causing mutations in both cultured human cells and animal models. Prime-editing systems have been used in preclinical studies to successfully treat genetic disorders such as sickle cell disease, retinitis pigmentosa and phenylketonuria [19,20,21]. The recent FDA approval for the first clinical trial of a prime-editing therapy for chronic granulomatous disease (CGD) further emphasizes the growing clinical potential of this technology.

Despite these significant advances, challenges remain, particularly in the clinical translation of the delivery of gene-editing tools to relevant tissues and cells in patients. Delivery methods that work in the laboratory or in animal models, such as viral vectors or lipid nanoparticles (similar to those used in mRNA COVID-19 vaccines), are being explored to improve the safety and efficacy of gene editing in humans. Additionally, ensuring that off-target effects such as unintended DNA changes are minimized remains a key focus of ongoing research and clinical development.

### 4.2. Next-Generation Precision Medicine Scientific, Regulatory and Global Access Challenges with a Focus on Sickle Cell Disease and Beta-Thalassemia, Tony Ho, MD, Pivotal Lifesciences

The success of ex vivo gene-editing therapies like exagamglogene autotemcel represents a significant breakthrough in the treatment of SCD and beta-thalassemia. However, the limitations of such therapies, including their high cost, complex manufacturing and need for myeloablation, restrict their global impact, particularly in LMICs [22]. Exagamglogene autotemcel, while life-changing for the small number of patients who can access it, is not scalable to the vast majority of individuals suffering from these diseases, especially in regions with limited healthcare infrastructure [23].

This brings into focus the urgent need for in vivo gene-editing therapies that are accessible, affordable and scalable. The ideal therapy would be a single IV administration that is curative and non-toxic and that can be delivered in all patient settings without the need for hospitalization and uses a vehicle that would only deliver to and only biologically activate at the cell of interest. The delivery vehicle must be non-immunogenic and low-toxicity. Despite significant progress in the development of delivery vehicles for gene therapy, particularly with the use of lipid nanoparticles (LNPs), most of the in vivo editing LNPs have targeted the liver (Table 2) [24].

Current research is exploring various non-viral delivery methods, such as targeted LNPs and viral-like particles (VLPs), to overcome the challenges of gene delivery beyond the liver, aiming to target specific cells like hematopoietic stem and progenitor cells (HSPCs) in SCD and beta-thalassemia [25]. Breda et al. demonstrated that antibodies to CD117 can be used as a targeting agent to deliver LNPs to HSPCs, achieving efficient gene editing with this drug delivery vehicle [26]. Similarly, Shi et al. has shown comparable results using the CD117 approach [27]. While in vivo gene editing offers an exciting pathway toward a global solution for genetic diseases, significant hurdles remain, including the need for precise delivery systems with low toxicity, mitigation of off-target effects, costs and pricing and the development of regulatory frameworks that ensure equitable access while maintaining incentives for pharmaceutical companies. Addressing these challenges through collaborative global efforts could enable the next generation of therapies to reach millions of patients worldwide, ultimately making gene-editing cures accessible to all.

### 4.3. Development of In Vivo Gene Therapy in Sickle Cell Disease, John Tisdale, MD, National Heart, Lung, and Blood Institute (NIH)

Advances in gene editing now present promising solutions, with tools such as lentiviral vectors and in vivo gene editing making the prospect of a more accessible and affordable one-time treatment increasingly realistic [28]. Lentiviral-based gene transfer has demonstrated significant results in clinical settings, notably reducing pain and hospitalizations for patients [29]. Current efforts focus on refining gene-editing techniques, particularly in vivo approaches, to overcome immune system barriers and efficiently target hematopoietic stem cells.

Targeting CD117, a marker on hematopoietic stem and progenitor cells (HSPCs), has been a central focus to advance non-chemotherapy-based conditioning. An antibody–drug conjugate has been developed to successfully ablate bone marrow without chemotherapy, which also has the advantage of preserving fertility post-treatment, as is being tested in animal models [30]. This progress opens up the possibility of leveraging antibody conditioning for gene-editing purposes, potentially expanding the conditions that could be targeted by genetic therapies by reducing off-target effects and favorably altering risk–benefit ratios.

The development of targeted delivery systems, including lipid nanoparticles, peptides, VLPs and viral vectors, offers new directions for precise therapeutic delivery to the bone marrow. Through comprehensive screening and optimization, promising candidates have been identified to enhance selectivity and efficacy in stem cell targeting. Despite these advancements, challenges remain in reaching enough hematopoietic stem cells to ensure lasting therapeutic benefits. Research estimates that approximately 100,000 hematopoietic stem cells actively generate blood at any given time, underscoring the need to target enough of these cells for successful gene therapies.

### 4.4. Adeno-Associated Viral (AAV) and Adenoviral (AdV) Vectors for In Vivo Gene Therapy, Hildegard Büning, PhD, Hannover Medical School

Recent advancements in viral vector systems, particularly adeno-associated viral (AAV) vectors, have paved the way for more precise and effective gene therapies. AAV, with its small, non-enveloped capsid, is capable of efficiently delivering DNA. However, challenges remain with its natural tropism, which results in a low efficacy in certain tissues following intravenous administration. These challenges are typically addressed by increasing the dosage of the vector. However, this often leads to immune system activation and other complications.

To address these limitations, researchers have adapted to engineering the AAV capsid through rational design and evolution-based techniques. By modifying specific regions of the capsid, it is possible to direct the vector to target cells, such as neurons, tumor cells or cells of the hematopoietic system, improving specificity and reducing off-target effects [31,32]. This approach not only enhances therapeutic efficacy but also minimizes the required dosage, potentially lowering production costs and reducing immune system complications.

Helper-dependent adenoviral vectors, larger than AAVs and with multiple serotypes, offer another promising vector system for gene therapy. These vectors are also engineered to improve tropism, facilitating the delivery of therapeutic genes to cells such as HSCs. A key strategy for utilizing adenoviral vectors is to mobilize hematopoietic stem cells, transduce them with modified vectors and then allow the cells to return to the bone marrow, where they can provide their therapeutic effects. This in vivo approach offers several advantages over ex vivo gene therapy, including reduced antigenic load, lower costs and simplified administration without the need for specialized centers or conditioning procedures.

Additionally, advancements in the development of next-generation vectors, including engineered AAVs and adenoviral vectors, allow for reduced immune responses. These improvements can extend treatment options to patients with pre-existing antibodies against the viral vectors and may enable repeated administrations. Engineering strategies also extend to the vector genome, optimizing its design for better stability, reduced immunogenicity and tailored transgene expression.

These innovations in AAV and adenoviral vectors are critical for moving them from ex vivo to in vivo applications and, ultimately, for their use as in vivo gene therapies for SCD. In vivo gene therapy promises greater accessibility, lower costs and a reduced time to treatment by eliminating the need for specialized centers and complex ex vivo manipulations. Ongoing research into vector engineering continues to push the boundaries of what is possible, moving toward more efficient and widely accessible gene therapies.

## 5. Session 4: Highlights from Discussion: Regulators’ Perspective

**Panelists: Sol Ruiz**, PhD, Spanish Medicines Agency (AEMPS); **Peter Marks**, MD, PhD, Center for Biologics Evaluation and Research, FDA; and **Eric Karikari-Boateng**, MS, Food and Drugs Authority Ghana.

Advances in gene therapies promise transformative treatments while also raising critical regulatory challenges. As these therapies move from small, targeted populations to broader applications, regulatory frameworks must evolve to address the varying risk–benefit profiles. The regulatory perspective highlights the need for robust oversight, international collaboration and coordination and product authenticity, especially in regions where healthcare infrastructure may be less developed.

***Key Highlights from this discussion***

- **Risk–Benefit Considerations for Different Populations:**

Regulatory scrutiny for gene therapies varies with the size of the target population. For small populations with severe or lethal genetic conditions, regulators may tolerate greater uncertainty due to the great potential benefits. In contrast, as gene therapies target larger populations, such as those for SCD, there will be a need for more robust safety and efficacy data to mitigate the risk of widespread adverse effects. Regulators must balance risk–benefit considerations, particularly as the size of the intended population grows and the seriousness and variability of the disease phenotype changes. In larger populations, there is a lower tolerance for uncertainty, necessitating extensive clinical and manufacturing data to ensure safety.

- **Ensuring Authenticity of Gene Therapy Products:**

A significant concern in gene therapy is ensuring the authenticity, reproducibility and quality of treatments, especially in regions prone to substandard or counterfeit products. Regulators must confirm that gene therapy products are legitimate, part of authorized clinical trials and meet stringent standards essential for patient safety and maintaining trust in the regulatory process.

- **Collaborative Regulatory Efforts:**

Global collaboration is crucial for converging regulatory requirements for gene therapies. Initiatives within the European Union focus on knowledge-sharing through workshops and training programs. Initiatives such as the African Medicines Agency are aimed at building regulatory capacity in developing regions. This collaborative approach was exemplified during the COVID-19 pandemic, where regulatory agencies worked together to accelerate decision-making.

Strengthening regulatory expertise in emerging regions like Africa is critical. Initiatives such as collaborative exchange programs are helping local regulators better assess gene therapy applications, thus ensuring a more robust oversight process.

Involving external experts from universities and hospitals is essential to navigate the complexities of gene therapies. These experts provide valuable insights that inform more effective regulatory decisions.

- **Early and Frequent Interaction with Developers:**

Ongoing engagement with developers from industry (both for-profit and not-for-profit) and academia is vital for regulatory success. Such interactions allow regulators to track development progress and provide guidance throughout the gene therapy development process.

- **Regulatory Frameworks:**

More agile regulatory structures will allow specialized groups to focus on areas like manufacturing and clinical review. This flexibility will expedite the development process while maintaining rigorous safety standards.

- **On-Demand Regulatory Advice:**

A new approach is needed to provide more accessible and real-time regulatory advice. This system enables developers to seek guidance without the need for formal meeting requests, potentially speeding up the development and approval of new therapies.

- **Global Convergence:**

There is a strong push for regulatory convergence across regions, particularly for rare disease gene therapies. Achieving global convergence could reduce the burden on developers and ensure wider access to innovative treatments.

## 6. Session 5: Highlights from Discussion: How Do We Prepare for the Next Generation of Gene Therapy, as the Industry, Regulators and a Healthcare System?

**Panelists: Hildegard Büning,** PhD, Hannover Medical School; **Cecelia Calhoun**, MD, MPHS, MBA, Yale University School of Medicine; **Jeremy Farrar**, MD, PhD, World Health Organization; **Julie Makani**, MD, PhD, Muhimbili University of Health and Allied Sciences (Tanzania), Tanzania High Commission to the UK; **Peter Marks**, MD, PhD, Center for Biologics Evaluation and Research, FDA; **Kwasi Nyarko**, PhD, WHO Regional Office for Africa (WHO-AFRO); and **Jimi Olaghere**, Gene Therapy Recipient.

The advancement of gene therapies presents a transformative opportunity for the treatment of conditions such as SCD. Achieving this potential requires robust collaborations across global regions among healthcare professionals, researchers and individuals living with the disease. The effective adoption of these therapies hinges on the establishment of strong healthcare delivery systems and investment in local infrastructure, particularly in underresourced regions. Innovations in treatment must be coupled with equitable access to ensure that advancements benefit diverse populations and address existing health disparities. A patient-led approach is essential as it prioritizes listening to patient experiences and involving them in decision-making processes. Furthermore, streamlining regulatory pathways and fostering bidirectional learning among stakeholders can enhance the deployment of gene therapies while promoting holistic care and economic viability. This commitment to community engagement, long-term monitoring and patient advocacy will be critical in advancing gene therapies and ultimately improving healthcare outcomes for all. The following key highlights from this panel discussion emphasize the multifaceted strategies and collaborative efforts necessary to realize the full potential of gene therapies in diverse healthcare settings.

- **Collaboration and Partnership:**

Emphasizing the necessity of partnerships across global regions to effectively implement gene therapies. Collaborative efforts among healthcare professionals, researchers and patients can enhance understanding and improve coordination of care delivery.

- **Healthcare Delivery Systems:**

Highlighting the importance of a robust healthcare delivery system to facilitate the administration of gene therapies. Current therapies only reach a small fraction of those who might benefit, signaling a need for infrastructural development and capacity-building, particularly in regions like Africa.

- **Innovation and Bidirectional Learning:**

Recognizing the innovative potential within global health contexts and the value of learning from international colleagues. Continuous multidisciplinary learning and collaborative partnerships can enhance understanding and improve deployment strategies for gene therapies.

- **Equity in Access to Therapies:**

Highlighting the importance of ensuring equitable access to gene therapies to prevent exacerbating global health inequalities. Proactive measures are necessary to ensure that scientific advancements benefit diverse populations, particularly marginalized communities.

- **Patient-led Approach:**

Underscoring the necessity of involving patients in the decision-making process and understanding their experiences. Comprehensive post-intervention and long-term care and support systems are critical for effective healthcare delivery.

- **Investment in Local Capacity:**

Emphasizing the need for investment in local healthcare to enable greater participation in gene therapy trials.

- **Accelerating Development Timelines and Streamlining Regulatory Processes:**

Highlighting the urgency of quicker development timelines for gene therapies, with a goal to deliver in vivo solutions within five years. Forming task forces with scientists, regulators and industry representatives can streamline regulatory pathways and ensure alignment on objectives.

- **Community Learning and Awareness:**

Acknowledging a shift in perspective towards valuing community learning, where advancements in one area can positively impact practices in others. Increasing awareness of gene therapy in regions like Africa is crucial for combating misinformation and fostering acceptance.

- **Regulatory Convergence and Future Preparedness:**

Noting the opportunity to establish convergence regulatory frameworks as gene therapy develops in Africa. Preparing regulatory frameworks for broader access and even coordination between countries and regulatory regions is essential while effectively managing current access.

Pointing out the need for regulators to balance rapid product availability while ensuring robust evidence for efficacy and safety, and to remain adaptable as new therapies emerge.

- **Capacity-Building Beyond Funding:**

Advocating for collaborative efforts, information exchange and ongoing partnerships as vital components of capacity-building, rather than relying solely on monetary investments.

- **Holistic Approach and Economic Viability:**

Emphasizing a holistic view in patient care, focusing on early identification and addressing patient needs to enhance outcomes. Setting economic expectations alongside clinical benefits ensures that gene therapies become financially viable for broader populations.

Highlighting the importance of long-term monitoring for gene therapy interventions to assess durability and safety. Collaboration between healthcare providers and patients is essential in determining monitoring preferences post-treatment.

Highlighting the real challenges faced by patients living with SCD, emphasizing the need for ongoing support and advocacy for improved treatment options.

- **Innovative Therapies and Diverse Treatment Options:**

Calling for innovation in both ex vivo and in vivo therapies, focusing on reducing manufacturing costs to improve accessibility. Recognizing the need for diverse treatment options for SCD patients beyond gene therapy to enhance their quality of life.

## 7. Conclusions

The advancement of gene therapies holds great promise. However, realizing their full potential requires collaborative efforts across regulatory bodies, healthcare systems and education of patient populations with diverse backgrounds. Equity in access to gene therapies emerged as a central theme, with the recognition that advancements in gene therapies must ideally be distributed fairly across populations to avoid exacerbating existing health disparities. Discussions highlighted the critical need for global collaboration in facilitating the successful implementation of and access to these therapies in regions limited in resources and with infrastructural gaps and regulatory challenges.

Proposed solutions presented to address the challenges to equitable access of gene therapies include bidirectional learning, community engagement and patient-centered approaches that prioritize long-term care. Additionally, investing in local capacity and building regional infrastructure were underscored as crucial components to support clinical trials and expand access to treatment, paired with efforts to sustain long-term follow-up. Equally as important is addressing cost barriers to ensure these innovative therapies are accessible to a wide range of patients. These approaches ensure that advancements in gene therapies are not only innovative but also accessible, acceptable and sustainable in diverse healthcare settings.

The sessions ultimately reaffirmed the need for a holistic approach—one that combines innovative therapies, supports patient advocacy and fosters an inclusive environment for advancing gene therapies. Achieving this vision will require extensive collaboration among regulatory bodies, patients and patient groups, the industry and healthcare providers to expand the benefits of gene therapy and address both immediate and long-term healthcare challenges worldwide.

## Figures and Tables

**Table 1:** FDA-approved gene therapies and CAR T-cell therapies.

Product Name	Indication	Type of Gene Therapy
Zolgensma	Spinal Muscle Atrophy	In vivo
Luxturna	Inherited Retinal Disease	In vivo
Skysona	Adrenoleukodystrophy	Ex vivo—gene addition
Zynteglo	Transfusion-dependent Thalassemia	Ex vivo—gene addition
Hemegenix	Hemophilia B	In vivo
Elevidys	Duchene’s Muscular Dystrophy	In vivo
Roctavian	Hemophilia A	In vivo
Casgevy	Sickle Cell Disease/Transfusion-dependent Thalassemia	Ex vivo—gene editing
Lyfgenia	Sickle Cell Disease	Ex vivo—gene addition
Lenmeldy	Metachromatic leukodystrophy (MLD)	Ex vivo—gene addition
Kymriah	Relapsed/Refractory B-cell Acute Lymphoblastic Leukemia (ALL) Relapsed/Refractory B-cell Non-Hodgkin’s Lymphoma (NHL)	Ex vivo—gene addition
Yescarta	B-cell Non-Hodgkin’s Lymphoma (NHL) Follicular Lymphoma	Ex vivo—gene addition
Tecartus	Mantle-cell Lymphoma (MCL) B-cell Non-Hodgkin’s Lymphoma (NHL)	Ex vivo—gene addition
Breyanzi	B-cell Non-Hodgkin’s Lymphoma (NHL)	Ex vivo—gene addition
Abecma	Multiple Myeloma	Ex vivo—gene addition
Carvykti	Multiple Myeloma	Ex vivo—gene addition

**Table 2:** In vivo hepatocyte editing using lipid nanoparticles—Currently in human clinical trials.

Company	Phase	Ongoing Research
Intellia Therapeutics	Phase 3	Utilizing CRISPR-based gene editing to target ATTR amyloidosis by editing the TTR gene
	Phase 2	Utilizing CRISPR-based gene-editing therapy for hereditary angioedema (HAE) by targeting the KLKB1 gene
Verve	Phase 1	Utilizing a gene-editing approach to lower cholesterol by targeting PCSK9
CRISPR Therapeutics	Phase 1	Conducting a Phase 1 clinical trial targeting atherosclerotic cardiovascular disease (ASCVD) through gene editing of the ANGPTL3 gene
	Phase 1	Conducting a Phase 1 clinical trial targeting atherosclerotic cardiovascular disease (ASCVD) by editing the gene responsible for lipoprotein (a)—Lp(a)

## Data Availability

Not applicable.

## Abbreviations

The following abbreviations are used in this manuscript:

AMA	African Medicines Agency
AdV	Adenoviral
AVAREF	African Vaccine Regulatory Forum
AAV	Adeno-associated viral
CAR T cell	Chimeric antigen receptor T cell
CGD	Chronic granulomatous disease
CRISPR	Clustered regularly interspaced short palindromic repeats
EMA	European Medicine Agency
GMP	Good manufacturing practice
HICs	High-income countries
HSCs	Hematopoietic stem cells
HSPC	Hematopoietic stem and progenitor
LMICs	Low-and middle-income countries
ML3	Maturity Level 3
IV	Intravenous injection
LNPs	Lipid nanoparticles
LMICs	Low-and middle-income countries
SCD	Sickle cell disease
SMA	Spinal muscular atrophy
U.S. FDA	United States Food and Drug Administration
VLPs	Viral-like particles
WHO	World Health Organization
